# War, Warriors, and Nurses

**Published:** 1916-07-22

**Authors:** 


### THE MATRONS' AND SISTERS' DEPARTMENT.

### WAR, WARRIORS, AND NURSES.

WE have had an interesting letter from a matron correspondent somewhere in France, who speaks in the highest terms of the first-aid base hospital and ambulance system and its working. She is full of admiration for the work done which moves patients and tends them so well that they arrive beautifully clean at the hospitals, with most carefully written notes fastened to their coats, and the wounded testify to the devotion of the ambulance men and their efficiency. She mentions that the prevailing feeling amongst the French patients seems to be that English nurses are better than French ones, but that the French surgeons are cleverer than the English. We should much like to have fuller information on these points, and to learn on what facts or experiences these opinions rest. The French soldier when wounded is a very amusing personage and deservedly liked in the hospital. He maintains an atmosphere of contented happiness throughout the day. Yet these men when they are asleep often cry out and utter sounds of distress which indicate that they are probably living over again some incident or incidents of their life in the trenches or at the Front. This makes a military hospital in France and elsewhere at the present time a place of surpassing interest for the anthropologist, the psychologist, and for real living ministers of God of all denominations. The latter can here obtain evidence which is supported by the two former, that this war has awakened the British and French soldier to his spirituality. He has put his religion into the war, and has so become one of the happiest, most joyous, most fearless, and wonderful types of humanity that the world throughout its history has ever had the good fortune to possess. The soldier is at peace with God. He lives from day to day, and is so without any shade or shadow of fear whilst he goes about God's business as a faithful soldier and patriot.

The war has materially changed the youth of the nation, and those nurses who have made their way to the Front are often changed, like the men, for the better. At the outset every woman, in proportion to her keenness, was anxious to go out to the war. What exactly were the reasons we need not consider, but, whatever they may have been, the best of the women who have returned after service abroad are found to have shed them by becoming grown up. Just as the young soldier left home as a youth and after service has returned as a man of settled convictions and sober judgment, so the nurse, by widening her atmosphere and being brought face to face with realities, has changed in so marked a manner as often to give her a presence and authority of which there was little or no trace before the war. War, therefore, has not been all drawback and trial, nor will it continue to be so, for it is capable of bringing many benefits, including higher standards of life, an accurate recognition of underlying causes, and for the workers a more kindly and cheerful outlook on life, its trials, teachings, and triumphs.

It is a great matter to learn on authority how beautifully kept the wounded are under the R.A.M.C. and ambulance period of their life at the Front. This means that time has enabled the perfecting of the organisation and the awakening of all workers in every branch of service who have to do with the wounded and the sick. We have little doubt that another outcome of the war will be the natural selection, resting upon evidence, of the women with administrative capacity which war service, ever changing and always varied, affords many opportunities to exhibit. We who are compulsorily retained at home may be cheered by realising these interesting facts and consequences. Another encouraging circumstance is the knowledge that the women workers at the Front who have had the advantage of education and are amongst the most capable of the nurses employed there, though often overworked and always busy, unconsciously reveal, like the wounded in their sleep, where their mind is engaged. We have evidence of this phase and consequence of the war in the receipt of schemes and suggestions by our nurses for improving the standard or increasing the quality of the work, or of the circumstances in which it is at present done in this country. It thus appears that the brighter minds amongst war workers in the nursing profession, who have awakened and developed through war-time, are moved with a keen desire to help their home sisters, to give them justice and opportunity for better circumstances and a higher standard of work, whoever and wherever they may be. One of the last expressed wishes of Lord Kitchener, as he was leaving for Russia, was "to see more religion put into the war by the people generally." Is this not one more proof of the wonderful man Lord Kitchener was, of his truth-penetrating power, and of the faint appreciation of his true character possessed by his contemporaries?

